# Comparison of different small molecular weight alcohols for sustaining sulfidogenic bioreactors maintained at moderately low pH

**DOI:** 10.3389/fbioe.2022.937987

**Published:** 2022-08-11

**Authors:** Ana Laura Santos, D. Barrie Johnson

**Affiliations:** ^1^ School of Natural Sciences, Bangor University, Bangor, United Kingdom; ^2^ Life Sciences, Natural History Museum, London, United Kingdom; ^3^ Health and Life Sciences, Coventry University, Coventry, United Kingdom

**Keywords:** acid, electron donors, ethanol, glycerol, methanol, sulfate-reducing bacteria, sulfidogenesis

## Abstract

Sulfate-reducing bacteria (SRB) catalyse the dissimilatory reduction of sulfate to hydrogen sulfide using a wide range of small molecular weight organic compounds, and hydrogen, as electron donors. Here we report the effects of different combinations of small molecular weight alcohols on the performance and bacterial composition of a moderately low pH sulfidogenic bioreactor (pH 4.0–5.5) operated at 35°C in continuous flow mode. Ethanol alone and methanol or ethanol used in combination with glycerol were evaluated based on their equivalent amounts of carbon. Although evidenced that methanol was utilised as electron donor to fuel sulfidogenesis at pH 5.5, rates of sulfate reduction/sulfide production were negatively impacted when this alcohol was first introduced to the system, though these rates increased in subsequent phases as a result of adaptation of the microbial community. Further increased dosage of methanol again caused rates of sulfidogenesis to decrease. Methanol addition resulted in perturbations of the bioreactor microbial community, and species not previously detected were present in relatively large abundance, including the sulfate-reducer *Desulfovibrio desulfuricans*. Ethanol utilization was evidenced by the increase in rates of sulfidogenesis as the dosage of ethanol increased, with rates being highest when the bioreactor was fed with ethanol alone. Concentrations of acetate in the effluent liquor also increased (up to 8 mM) as a result of incomplete oxidation of ethanol. This alcohol continued to be used as the electron donor for sulfate reduction when the bioreactor pH was decreased incrementally (to pH 4.0), but rates of sulfidogenesis decreased. The relative abundance of *Dv. desulfuricans* diminished as the bioreactor pH was lowered, while that of the acidophilic *Firmicute Desulfosporosinus acididurans* increased. This study has shown that all three alcohols can be used to fuel microbial sulfidogenesis in moderately acidic liquors, though the cost-effectiveness, availability and toxicity to the microbial community will dictate the choice of substrate.

## Introduction

Generation of acid mine drainage (AMD) from metal and coal mining is one of the most serious contaminants of water bodies around the world. Base metals of economic importance in environmentally-benign generation and storage of energy, such as cobalt, nickel, manganese, etc. Can be present in these mining-impacted waters, in some cases in concentrations high enough to make their recovery economically feasible. Microbial treatment of AMD by sulfate-reducing bacteria (SRB) has considerable advantages over conventional remediation strategies since the metal sulfides generated have relatively high densities, are highly insoluble and can allow chalcophilic metals to be selectively recovered and recycled. Sulfate-reducing bacteria can be found in both natural and anthropogenic environments where sulfate is present, such as marine and some freshwater sediments, and some industrial wastewaters. These microorganisms catalyse the dissimilatory reduction of sulfate (or elemental sulfur) generating hydrogen sulfide (H_2_S/HS^−^) using mostly small molecular weight organic compounds as electron donors (Costa et al., 2021), though some can also use hydrogen (Lens et al., 2003). More than one hundred organic compounds have been reported to serve as electron donors for SRB which grow at circum-neutral pH, including carbohydrates (e.g., fructose and glucose), alcohols (e.g., ethanol, methanol), amino acids (e.g., serine, alanine), monocarboxylic acids (e.g., acetate, propionate), dicarboxylic acids (e.g., fumarate, succinate), aromatic compounds (e.g., phenol) (Liamleam and Annachhatre, 2007; Lakaniemi et al., 2010; Cao et al., 2012; Hussain et al., 2016). Lactate is frequently used to enrich and cultivate neutrophilic SRB and has been demonstrated to be effective for remediating some metal-rich contaminated wastewaters (Widdel, 1988; Hammack et al., 1994). AMD usually contains low concentrations of organic compounds which therefore need to be added (e.g., in composts in passive remediation systems). The choice of substrate(s) used depends mainly on local availability and costs (Sánchez-Andrea et al., 2014). The potential toxicity of substrates also needs to be taken into account, particularly in low pH biosulfidogenic systems where many organic acids, including lactate and acetate, are present predominantly in their undissociated forms that can permeate through cell membranes, causing the acid to dissociate in the neutral pH cytoplasm, releasing protons and causing the internal pH to decrease, severely impacting metabolic activities, and possibly causing cell death. Therefore, the choice of substrate used in acidophilic biosulfidogenic processes is crucial, and non-ionic substrates are usually more appropriate for enriching and cultivating SRB at low pH (Hard and Babel, 1997; Sen and Johnson, 1999). Kimura et al. (2006) have described an alternative solution based on microbial syntrophic interactions whereby the heterotrophic, non-sulfidogen *Acidocella aromatica* strain PFBC consumed the acetic acid produced during the incomplete oxidation of glycerol by the sulfate-reducer *Desulfosporosinus* (*D.*) *acididurans*, eliminating the issue of acetic acid toxicity in low pH sulfidogenic bioreactors.

Suitable organic substrates that are economically sustainable, locally produced, or easily transported and stored, are key factors for the feasibility of treating mine water using SRB bioreactors (Bomberg et al., 2017). Ethanol can fulfil all aforementioned requirements and has successfully been applied in circum-neutral pH biosulfidogenic processes for remediating AMD, such as the first full-scale plant used to remediate acidic zinc-contaminated groundwater at the Nyrstar zinc refinery at Budel-Dorplein, Netherlands (Muyzer and Stams, 2008). Glycerol has been used routinely as the carbon- and energy-source for acidophilic SRB in bioreactor modules used to remediate AMD (Ñancucheo and Johnson, 2012; Hedrich and Johnson, 2014; Santos and Johnson, 2017). Both ethanol and glycerol can either be oxidised completely (to CO_2_) or incompletely (generating both acetate and CO_2_) by SRB, as shown in reactions (1–4):

$$4\;{\text{C}}_{3}{\text{H}}_{8}{\text{O}}_{3}+7\;{\text{SO}}_{4}^{2-}+14\;{\text{H}}^{+}\to 12\;{\text{CO}}_{2}+7\;{\text{H}}_{2}\text{S}+16\;{\text{H}}_{2}\text{O}$$ (1)

$$4{\text{ C}}_{3}{\text{H}}_{8}{\text{O}}_{3}+3\;{\text{SO}}_{4}^{2-}+2\;{\text{H}}^{+}\to 4\;{\text{CH}}_{3}{\text{COO}}^{-}+4\;{\text{CO}}_{2}+3\;{\text{H}}_{2}\text{S}+8{\text{ H}}_{2}\text{O}$$ (2)

$${2\text{ C}}_{2}{\text{H}}_{5}\text{OH }+\;3\;{\text{SO}}_{4}^{2-}{+\;6\text{ H}}^{+}\to {4\text{ CO}}_{2}{+\;3\text{ H}}_{2}{\text{S }+\;6\text{ H}}_{2}\text{O}$$ (3)

$${2\text{ C}}_{2}{\text{H}}_{5}{\text{OH}+\text{SO}}_{4}^{2-}\to {2\text{ CH}}_{3}{\text{COO}}^{-}{+\text{ H}}_{2}{\text{S}+2\text{ H}}_{2}\text{O }$$ (4)

Incomplete substrate oxidation has a negative impact on the performance of a sulfidogenic bioreactor as it causes less sulfate to be reduced/electron donor oxidised compared to complete substrate oxidation. Methanol has been reported to be used as an electron donor by some SRB (e.g., Braun and Stolp, 1985; Hard and Babel, 1997) and this C1 alcohol is oxidised to CO_2_ only (reaction 5):

$${4\text{ CH}}_{3}{\text{OH}+3\text{ SO}}_{4}^{2-}{\;+6\text{ H}}^{+}\to {4\text{ CO}}_{2}{+3\text{ H}}_{2}{\text{S }+8\text{ H}}_{2}\text{O}$$ (5)

Glycerol, in crude form, is produced in large quantities as a by-product of biodiesel production (Leoneti et al., 2012). This material can contain high concentrations of methanol, which is used in excess during the transesterification of vegetable oils and animal fat and is not recovered. Though numbers are highly variable, crude glycerol typically contains (w/w): 30% glycerol, 50% methanol, 13% “soap”, 2% moisture, 2–3% salts and 2–3% other impurities (Rehman et al., 2008). Using this by-product as a carbon/energy source for sulfidogenic bioreactors rather than pure glycerol would reduce operational costs.

This work described laboratory experiments in which the effects of different combinations of methanol or ethanol and glycerol as substrates for a moderately low pH (4.0–5.5) sulfidogenic bioreactor were assessed, both in terms of the performance of the bioreactor and the composition of the indigenous biomass.

## Materials and methods

### Sulfidogenic bioreactor

**FIGURE 1:**
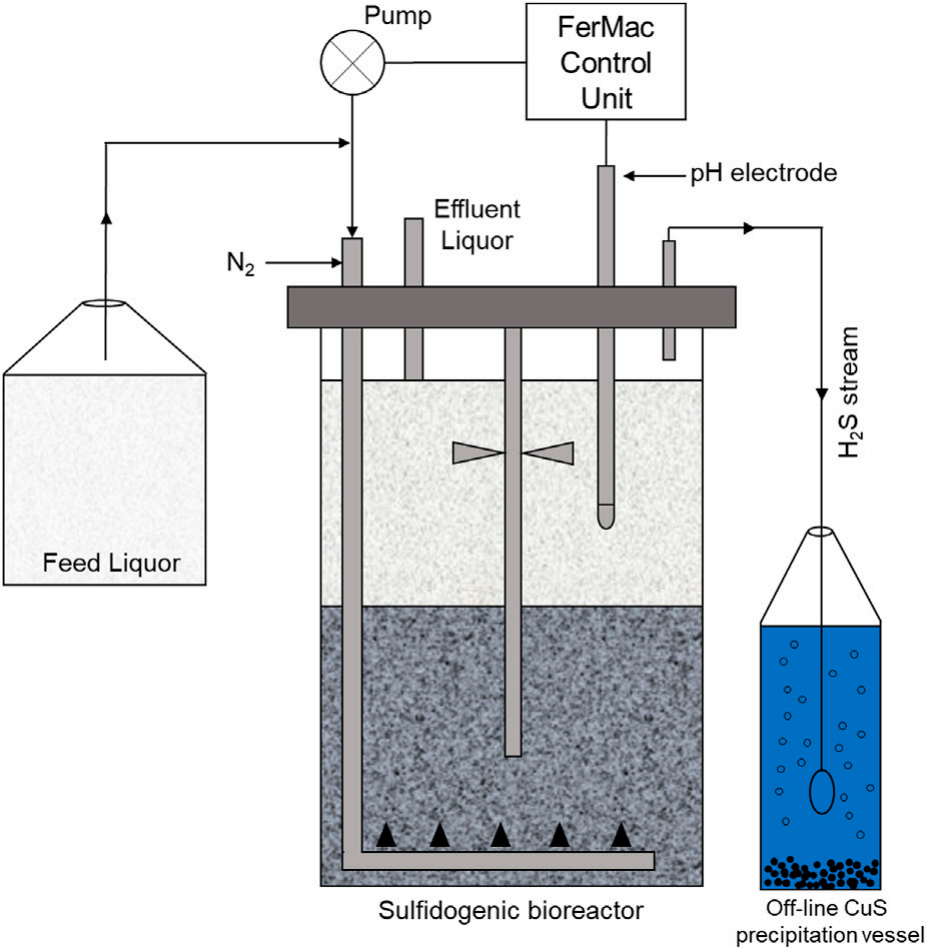
Schematic representation of the low pH sulfidogenic bioreactor. The arrows indicate the direction of liquid/gas flow Santos and Johnson (2017).

A sulfidogenic up-flow biofilm reactor, based on a similar system described by Ñancucheo and Johnson (2012) was used in the experimental work (Figure 1). The bioreactor contained several species of acidophilic SRB and non-sulfidogenic bacteria immobilized on 1–2 mm diameter porous beads manufactured from recycled glass (Dennert Poraver GmbH, Schlűsselfeld, Germany). The bioreactor had a working volume of 2.0 L and was coupled to a FerMac 310/60 control unit (Electrolab Biotech, United Kingdom). A pH electrode (Broadley James, United Kingdom), located in the liquid phase above the biofilm bed was coupled to the acid-input pump of the control unit. This pump controlled the flow the influent liquor into the reactor, which was added at a variable rate, required to maintain the pH of the bioreactor liquor at any set value. Influent liquor was pumped into the bottom of the bioreactor vessel via a sparger tube percolated upwards through the biofilm bed into the overlying liquid layer which was stirred gently at 50 rpm in order not to disrupt the biofilm bed. Flow rates were dictated by the pH differential of the influent liquor (pH 2.5) and the set operating pH of the bioreactor (pH 5.5). A drain tube placed above the liquid surface coupled to a second pump on the control unit ensured that the liquid volume within the bioreactor remained constant. The temperature of the bioreactor was set at 35°C. Oxygen-free nitrogen (OFN) was continuously gassed through the bioreactor at 150 ml min^−1^, both to promote anoxic conditions within the vessel and to act as a carrier gas to remove and transfer H_2_S generated in the bioreactor to an off-line vessel containing 400 ml of 25 mM copper sulfate. Feed liquor contained basal salts and trace elements (Ñancucheo et al., 2016) and 100 µM ferrous sulfate, was supplemented with 2.5 mM MgSO_4_ and 0.1 g L^−1^ yeast extract and pH adjusted to 2.5 with sulfuric acid. Concentrations of electron donors (ethanol alone and methanol or ethanol used in combination with glycerol) are described below.

### Operational set up of the sulfidogenic bioreactor using combined methanol and glycerol as potential electron donors

The sulfidogenic bioreactor was operated in continuous flow mode for 132 days using variable combinations of methanol and glycerol as potential substrates. The effect of methanol on the performance and composition of the SRB bioreactor was evaluated by combining different concentrations of methanol and glycerol in the feed liquor. The relative percentages of methanol and glycerol were based on the relative amounts of carbon they contain [methanol is a C_1_ compound while glycerol is a C_3_ compound (Table 1)]. Phases of the experiment are referred to as M0, M10, M25, M50 and M75, referring to the relative amounts (in terms of carbon equivalents; 0–75%) of methanol present in the influent liquor. No tests were carried out using 100% methanol in the feed liquor.

**TABLE 1:** Ratios of methanol and glycerol in the influent liquors and their combined carbon equivalents. An influent liquor containing only methanol was not tested.

Phases	Methanol (relative %)	Methanol (mM)	Glycerol (mM)	Total carbon equivalent (mM)	Test duration (days)
M0	0	0	5.0	15	6
M10	10	1.5	4.5	15	46
M25	25	4.0	4.0	16	35
M50	50	8.0	2.5	16	21
M75	75	12.0	1.0	15	24

### Ethanol as an electron donor for sustaining low pH biosulfidogenesis

The effect of ethanol as electron donor on the performance and composition of the sulfidogenic bioreactor was assessed over a period of 212 days. The bioreactor was operated initially at pH 5.5 and 35°C and fed with the medium described above, and containing different combinations of ethanol and glycerol in the feed liquor (Table 2). Concentrations of ethanol were increased, and glycerol decreased progressively until ethanol was the sole electron donor in the influent liquor. Once the bioreactor performance had become relatively stable using only ethanol as electron donor, its pH was gradually lowered from 5.5 to 4.0 in order to evaluate the effect of low pH ethanol-fuelled biosulfidogenesis. The phases referred to in Table 2 and the text below refers to the substrate used (E; ethanol) and its relative percentage (compared to glycerol, as carbon equivalents: 0–100) and the pH value at which the bioreactor was operated.

**TABLE 2:** Ratios of ethanol and glycerol in the influent liquors and their combined carbon equivalents.

	Ethanol (relative %)	Ethanol (mM)	Glycerol (mM)	Total carbon equivalent (mM)	Bioreactor pH	Test duration (days)
E0	0	0	5.0	15	5.5	17
E10	10	1.0	4.5	16	5.5	33
E25	25	2.5	3.5	16	5.5	48
E50	50	5.0	2.5	16	5.5	12
E75	75	6.5	1.0	16	5.5	10
E100_5.5	100	8.0	0	16	5.5	10
E100_5.0	100	8.0	0	16	5.0	20
E100_4.5	100	8.0	0	16	4.5	20
E100_4.0	100	8.0	0	16	4.0	42

### Analytical techniques

Liquid samples draining the bioreactor were removed at regular intervals and filtered through 0.2 µm nitro-cellulose membrane filters (Whatman, United Kingdom). Concentrations of sulfate and acetate were determined using a Dionex IC25 ion chromatograph with an Ion Pac AS-11 column equipped with a conductivity detector. Glycerol was determined using a Dionex ICS 3000 ion chromatography system fitted with a Carbo Pac MA1 column and ED amperometric detector. Data were analyzed using the Chromeleon® software package (Dionex, U.S.A.). Concentrations of methanol and ethanol in influent and effluent liquors were determined using enzymatic assay described by Vinet (1987) and Bostick and Overton (1980), respectively. For the methanol assay, 200 μl enzyme reagent (containing 1.8 ml of 2.5 mM NAD^+^ and 0.2 ml formaldehyde dehydrogenase solution prepared at 5 U mL^−1^) and 20 μl alcohol oxidase solution (5 U mL^−1^) were added into 1.5 ml centrifuge tubes and incubated at 30°C for 5 min. Then, transferred to a 96-well UV-star flat bottom microplate (Greiner Bio-one, United Kingdom) to which 50 μl of sample was added. Samples were analysed using a Multiskan™ GO Microplate Spectrophotometer (Thermo Fisher Scientific, United Kingdom). For the ethanol assay, 200 μl enzyme reagent (containing 1.8 ml of 2.5 mM NAD^+^ and 0.2 ml alcohol dehydrogenase solution prepared at 5 U mL^−1^ and 15 μL 10% hydrazine solution) was added into 1.5 ml centrifuge tubes and incubated at 30°C for 5 min. Samples were added (50 μL) and mixed thoroughly. The total volume was transferred to a 96-well UV-star flat bottom microplate and analysed as described above. In both assays, readings were carried out at 340 nm, with linear shaking for 5 s prior to analysis. Changes in absorbance were measured over a 10 min period for methanol and 20 min period for ethanol (1 reading min ^−1^).

Hydraulic retention times (HRTs), expressed in hours (h), were calculated by dividing the working volume of the bioreactor by flow rates. Rates of sulfate reduction (as mmol SO_4_^2-^ reduced L^−1^ day^−1^) were calculated from differences in concentrations of sulfate in inflow minus outflow liquors (sulfate consumption) and the actual flow rates (L day^−1^). Rates of hydrogen sulfide production were calculated from rates of off-line copper precipitation (H_2_S + Cu^2+^ → CuS + 2H^+^). Concentrations of soluble copper were monitored regularly in the off-line vessel using a colorimetric-based assay (Anwar et al., 2000), and corresponding rates of H_2_S production expressed as mmol L^−1^ day^−1^.

### Molecular analyses of the planktonic bacterial population in the bioreactor

Liquid samples were removed from between 2–5 cm above the biofilm bed at the end of each phase to assess the compositions of the planktonic bacterial populations. DNA was extracted using PowerSoil Ultraclean Microbial DNA Isolation Kit (MoBio, CA, United States). Bacterial 16S rRNA genes from extracted DNA were amplified for terminal restriction enzyme fragment length polymorphism (T-RFLP) using a modified Cy5-labeled 27f primer (5′-AGT GTT TGA TCC TGG GTC AG-3′) and unlabeled 1387r (5′- GGG CGG WGT GTA CAA GGG-3′). The purified products were digested separately in a 10 µL reaction containing 1U of restriction endonuclease HaeIII, CfoI and AluI (Promega, United Kingdom) and 1 µl of appropriate 10X buffer in each reaction tube. The reactions were incubated at 37°C for 1 h. Restriction fragments (T-RFs) were analysed on a capillary sequencer (Beckman Coulter, CEQ8000) and identified by comparison to the database of acidophilic microorganisms held at Bangor University. Relative abundance of T-RF’s were calculated on the basis of peak areas. Clone libraries were constructed to identify unknown organisms detected in T-RFLP analyses (Falagán et al., 2014). Theoretical T-RFs of the clones were obtained by measuring the lengths of the bacterial 16S rRNA genes fragments cleaved by the restriction enzyme HaeIII and compared to that obtained from T-RFLP analysis.

## Results

### Effects of different combinations of methanol and glycerol on the performance of the sulfidogenic bioreactor and molecular analyses of the planktonic bacterial population

**FIGURE 2:**
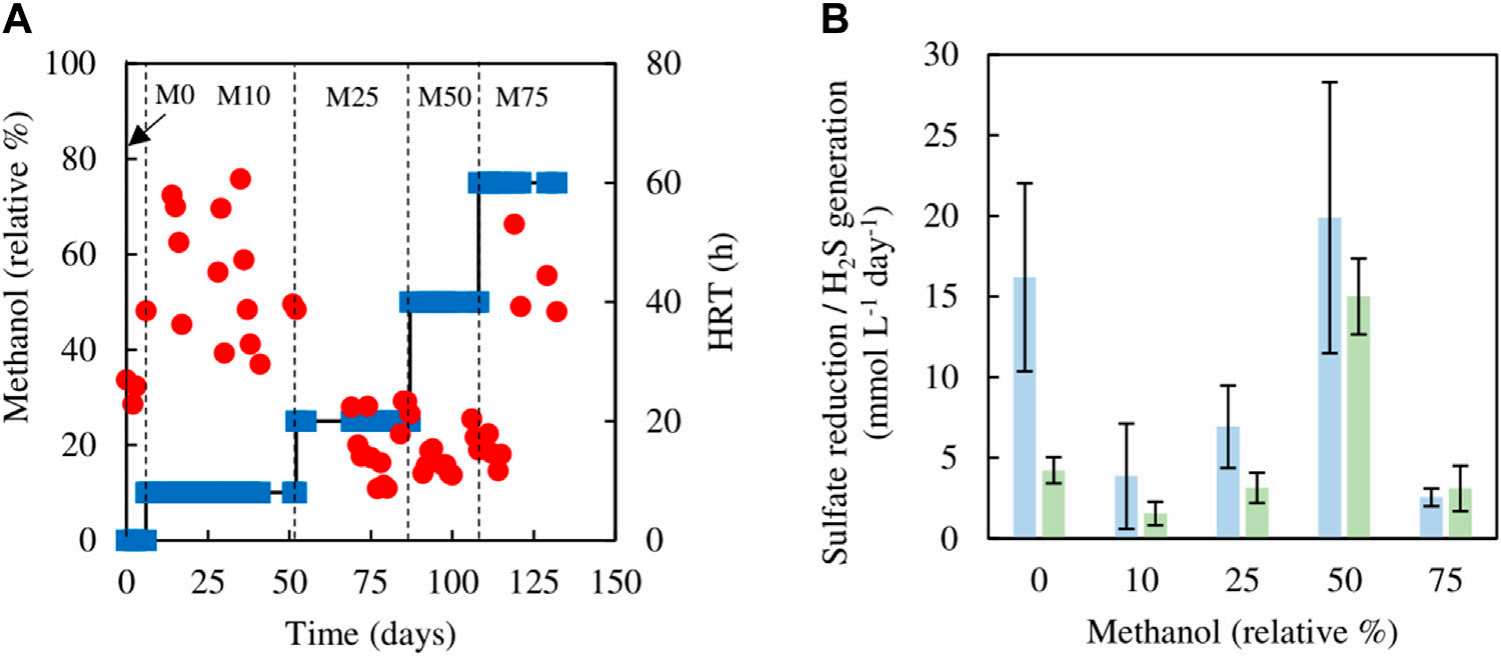
**(A)** Changes in HRTs (●) with varying relative percentages of methanol (■) used in the feed liquor of the sulfidogenic bioreactor, and **(B)** variations in rates of sulfate reduction (blue bars) and hydrogen sulfide generation (green bars) in each combination of methanol and glycerol tested (*n* = 11).

The sulfidogenic bioreactor, which had been previously fed with media containing only glycerol (and yeast extract) for over a year, was tested initially using different combinations of glycerol and methanol. Changing from 5 mM glycerol to 1.5 mM methanol/4.5 mM glycerol (M10) resulted in an immediate increase in HRT from 23 to 60 h, which was followed by a decrease on day 17, though HRTs varied greatly between 30 and 60 h through phase M10 (day 7–52) (Figure 2A). Hydraulic retention times were lower (varying between 8 and 25 h) during phases M25 (day 55–87) and M50 (day 91–108). During phase M75 (day 109–132), HRTs increased again, reaching up to 53 h (Figure 2A).

Figure 2B shows both rates of sulfate reduction calculated from differences in influent and effluent sulfate concentrations, and H_2_S generation based on off-line precipitation of CuS. These were both subject to considerable variation, but apart from phase M0 (zero methanol) they were not significantly different to each other. Both of these measurements of sulfidogenesis declined in phase M10 when methanol was first introduced, and these changes correlated with the observed large increases in HRTs. Thereafter (phases M25 and M50), rates of sulfidogenesis increased in line with lower HRTs, though when the amount of methanol in the feed liquor was increased further (M75), sulfidogenesis again declined, corresponding to increased HRTs. The effect of using methanol as sole electron donor on sustaining biosulfidogenesis was not evaluated to avoid accentuating the negative impact, suggested by the dramatic decrease in both measurements of sulfidogenesis in phase M75.

**FIGURE 3:**
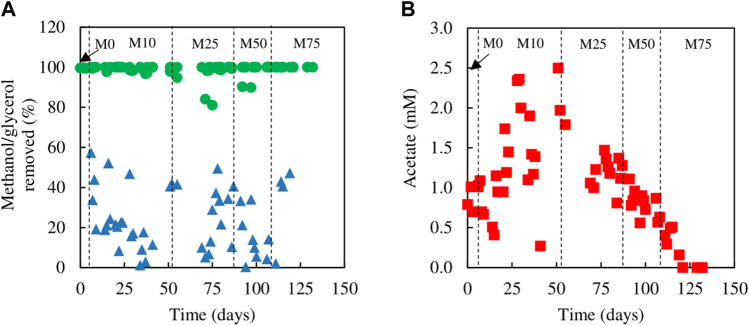
**(A)** Differences in concentrations of **(A)** methanol (▲) and glycerol (●) removed, and **(B)** concentrations of acetate (■) present in effluent liquors, during tests carried out with mixtures of glycerol and methanol in the feed liquor.

Evidence that methanol was being used as electron donor coupled to sulfate reduction was obtained from measurements of SRR and H_2_S production, and concentrations of glycerol, methanol and acetate in influent and effluent liquors (Figure 3), with reference to the stoichiometries of the reactions shown in Eqs 1, 2, 5. While concentrations of glycerol in the effluent liquors were generally only about 1% of those in the influent liquors, corresponding values for methanol were generally ∼80%, though were sometimes as low as ∼50%. Acetate (a product of incomplete oxidation of glycerol) was detected throughout, with concentrations being highest in phases M10 and M25 and declining when the amount of glycerol in the feed liquor was reduced in phases M50 and M75. Analytical data from phase M75 confirmed that methanol was being used, in part, to fuel sulfidogenesis. For example, at one time the difference in concentrations of glycerol entering and leaving the bioreactor was 0.95 mM, and that of acetate in the effluent liquor was 0.2 mM, implying that 0.75 mM glycerol was oxidised completely, which would have accounted for a maximum of 1.3 mM sulfate reduced (Eq. 1), and 0.2 mM was oxidised incompletely, which would have accounted for 0.2 mM sulfate reduced (Eq. 2), totalling 1.5 mM sulfate reduced. The corresponding difference in sulfate concentrations (influent and effluent liquors) on that occasion was much greater (4.7 mM), which was assumed primarily due to methanol acting as a second electron donor for sulfate reduction at that time. Around 50% of the methanol was removed (∼6 mM) which could have accounted for an additional 4.5 mM sulfate reduction (Eq. 5).

**FIGURE 4:**
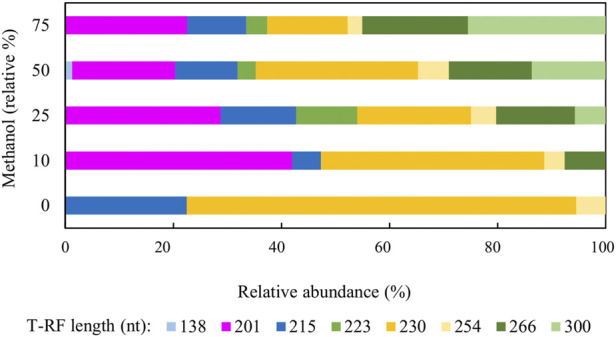
Terminal restriction enzyme fragment length polymorphism (T-RFLP) analysis (HaeIII digests) of amplified 16S rRNA genes of planktonic bacterial communities in the sulfidogenic bioreactor fed with different combinations of methanol and glycerol. Shades of blue represent known sulfate-reducing *Firmicutes* (138 nt, *Peptococcaceae* CEB3; 215 nt, *D. acididurans*), shades of yellow represent non-sulfidogens (230 nt, *Actinobacterium* AR3; 253 nt, *At. ferrooxidans*), shades of green represent bacterial clones identified as probable non-sulfidogens, while the magenta bars represent clone SRB7, which was positively identified as a strain of the sulfate-reducer *Dv. desulfuricans*.

Figure 4 shows changes in relative abundance of members of the planktonic bacterial community of the sulfidogenic bioreactor when both glycerol and methanol were added to the feed liquor. In phase M0 the bacterial community was dominated by the *Actinobacterium* AR3 (230 nt; Santos and Johnson, 2017) which accounted for ∼70% of the planktonic population in the bioreactor. The only sulfate-reducing bacterium detected during this phase was *D. acididurans* ^T^ (215 nt) (Sánchez-Andrea et al., 2015), which accounted for ∼22% of the relative abundance of the bacterial population. The facultative anaerobic bacterium *Acidithiobacillus* (*At.*) *ferrooxidans* (254 nt) was also detected during this phase (∼5%). Addition of methanol to the feed liquor caused major changes in the microbial population of the bioreactor (Figure 4). In phase M10, two unknown T-RFs (201 nt and 266 nt) were detected which together accounted for almost 50% of the planktonic bacterial community. In phases M25, M50 and M75, these unknown T-RFs were again detected, plus two others (223 nt and 300 nt). Together, these accounted for 60–75% of the total planktonic bacterial T-RFs in the bioreactor. Interestingly, the 138 nt T-RF, which corresponded to the sulfate-reducer *Peptococcaceae* CEB3 was only detected in phase M50, at very low relative abundance (∼2%).

The identities of the clones from a clone library constructed based on the biomass harvested at the end of phase M75 are shown in Table 3. Most of the bacterial clones obtained were closely related to organisms already described at species level, and their theoretical T-RFs matched those found in T-RFLP analyses. For example, clones SRB3, SRB15, and SRB19 were 99% related to *Clostridium beijerinckii*, a genus known to include species that ferment glycerol and other substrates in anaerobic systems (Sanguanchaipaiwong and Leksawasdi, 2017). Clone SRB7 was 99% related to *Desulfovibrio desulfuricans* which is a known neutrophilic sulfate-reducing bacterium. The presence of another SRB in the system would explain the higher rates of sulfate reduction obtained during phases M25 and M50.

**TABLE 3:** Identity of cloned bacterial 16S rRNA genes obtained from the sulfidogenic bioreactor (*n* = 19).

Clone designation	Closest relative	% Identity (16S rRNA gene)	Theoretical T-RF (nt) (HaeIII)	References
SRB3 (*n* = 2)	*Clostridium beijerinckii* NRRL B-598 (CP011966.2)	99	302	Unpublished
SRB7 (*n* = 4)	*Desulfovibrio* (*Dv.*) *desulfuricans subsp. desulfuricans* strain Essex 6 (NR_104990.1)	99	201	Loubinoux et al. (2000)
SRB8 (*n* = 3)	*Bacteroides caecigallinarum* strain C13EG153 (AB861982.1)	89	266	Saputra et al. (2015)
SRB11 (*n* = 5)	*Propionicimonas paludicola* strain Wd (NR_104769.1)	99	223	Akasaka et al. (2003)
SRB15 (*n* = 2)	*Clostridium beijerinckii* strain BAS/B3/I/124 (CP016090.1)	98	300	Unpublished
SRB19 (*n* = 3)	*Clostridium beijerinckii* isolate DSM 6423 (LN908213.1)	99	300	Unpublished

### Effects of different combinations of ethanol and glycerol on the performance of the sulfidogenic bioreactor and molecular analyses of the planktonic bacterial population

**FIGURE 5:**
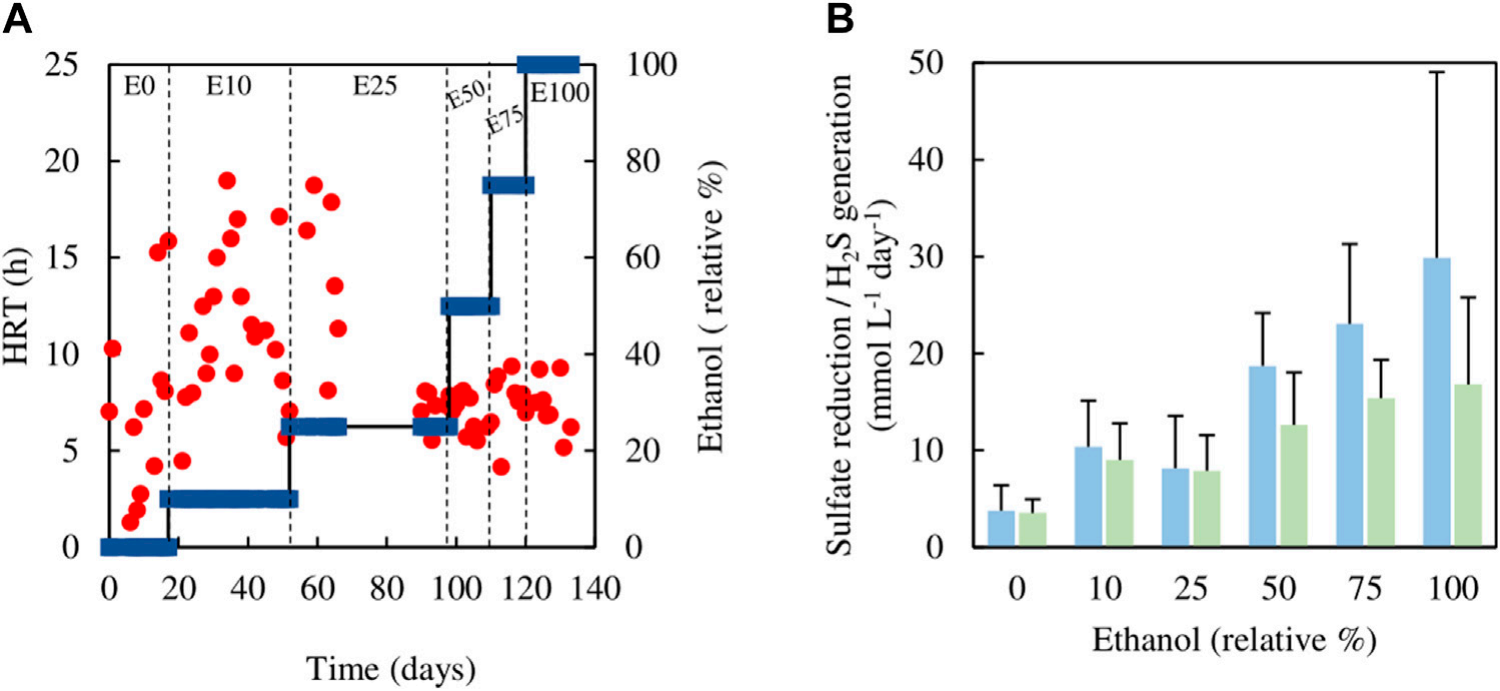
**(A)** Changes in HRTs (●) with different relative percentages of ethanol (■) present in the feed liquor of the sulfidogenic bioreactor, and **(B)** variations in rates of sulfate reduction (blue bars) and hydrogen sulfide generation (green bars) in each combination of ethanol and glycerol tested (*n* = 15). Samples were not analysed in the interval between day 66 and day 90.

**FIGURE 6:**
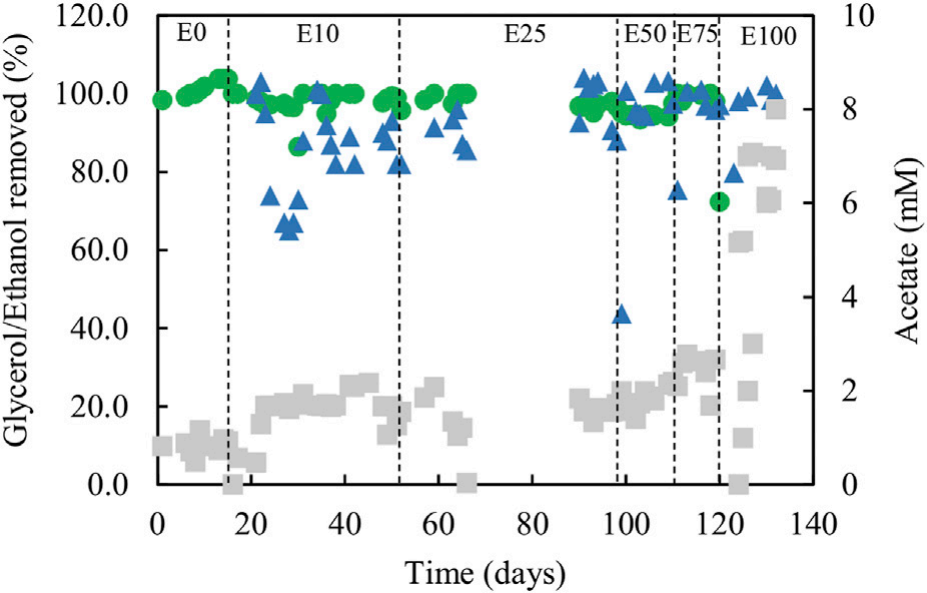
Differences in concentrations of ethanol (▲) and glycerol (●) removed, and concentrations of acetate (■) in effluent liquors, during tests carried out with mixtures of glycerol and ethanol in the feed liquor. Samples were not analysed between days 66 and 90.

Analytical data from the sulfidogenic bioreactor when fed with different ratios of ethanol and glycerol as principle electron donors are shown in Figures 5, 6. There were large fluctuations in HRTs during the first 66 days of the experiment (phases E0, E10 and the first part of E25), though later these became much more stable (phases E50, E75 and E100; Figure 5A).

Estimates of sufidogenesis from SRR and H_2_S production were generally well correlated, though means values of the former were generally greater than those of the latter (Figure 5B). There were progressive increases in average rates of SRR and H_2_S production when relative concentrations of ethanol in the feed liquor were increased (Figure 5B). Average rates of sulfate reduction rates varied between 4.0 mmol L^−1^ day^−1^ in phase E0 to ∼30 mmol L^−1^ day^−1^ during phase E100. Figure 6 shows that >99% of the glycerol was removed, as was 65–99% of the ethanol present in the influent liquor during all operational phases. However, relatively high concentrations of acetate were produced, particularly in phase E100, where they approached 8 mM (Figure 6). This implied that most, if not all of the ethanol was being incompletely oxidised. From Eq. 4, this would allow the reduction of ∼8 mM sulfate to sulfide, and this was supported by analytical data which showed that about 9 mM sulfate was reduced during phase E100.

**FIGURE 7:**
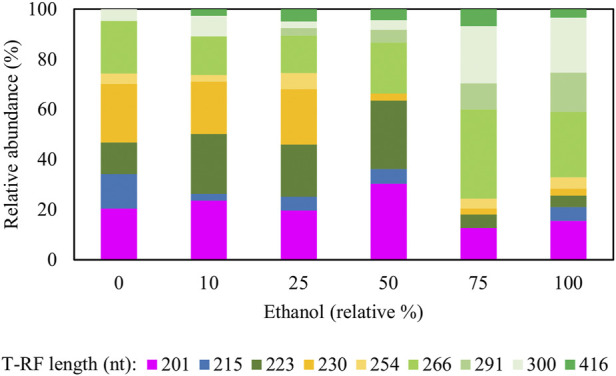
Terminal restriction enzyme fragment length polymorphism (T-RFLP) analysis (HaeIII digests) of amplified 16S rRNA genes of planktonic bacterial communities in the sulfidogenic bioreactor fed with different combinations of ethanol and glycerol. Shades of blue represent known sulfate-reducing *Firmicutes* (215 nt, *D. acididurans*), shades of yellow represent non-sulfidogens (230 nt, *Actinobacterium* AR3; 253 nt, *At. ferrooxidans*), shades of green represent bacterial clones identified as probable non-sulfidogens and the magenta bars represent clone SRB7 (201 nt; *Dv. desulfuricans*).

Figure 7 shows changes in relative abundance of members of the planktonic bacterial community of the sulfidogenic bioreactor when either or both ethanol and glycerol were present in the feed liquor. In phase E0, the most abundant bacterium was the *Actinobacterium* AR3 (230 nt), accounting for ∼25% of the relative abundance of the bacterial population. Two sulfate-reducing bacteria were detected during this phase, *D. acididurans* ^T^ (215 nt) and *Dv. desulfuricans* represented by clone SRB7 (201 nt; 99% gene similarity) and accounted for ∼35% of the total bacterial population. The facultative anaerobic bacterium *At. ferrooxidans* (254 nt) was also detected in this phase (∼4%) plus the clones whose T-RFs matched those found in the T-RFLP analyses (Table 3).

Addition of ethanol to the feed liquor caused major shifts in the microbial population of the bioreactor (Figure 7). In both phases E10 and E25, the T-RFLP profiles were similar to that obtained at phase E0, though an additional T-RF (291 nt) was detected in E25 in low (∼0.3%) relative abundance. Two SRB (*D. acididurans* ^T^ and *Dv. desulfuricans*) were detected in all phases with relative abundance varying between 15% (in E75) and 35% (in E50). A 416 nt length T-RF, which had not been detected in previous experiments, was present in DNA extracted from all effluents when ethanol was provided, though in low relative abundance. The 223 nt, 300 nt, 266 nt and 201 nt T-RFs corresponded to clones found in the clone library analysis described earlier. Two other new T-RFs were detected (291 nt and 416 nt), but no further analyses were carried out on these. The T-RFLP profile also shows a major shift from phases E0 - E50 to phases E75 - E100, where only ∼10–20% of the planktonic bacterial population were known SRB.

### Ethanol as an alternative electron donor to glycerol for sustaining sulfidogenesis at pH 4.0–5.5 and molecular analyses of the planktonic bacterial population

**FIGURE 8:**
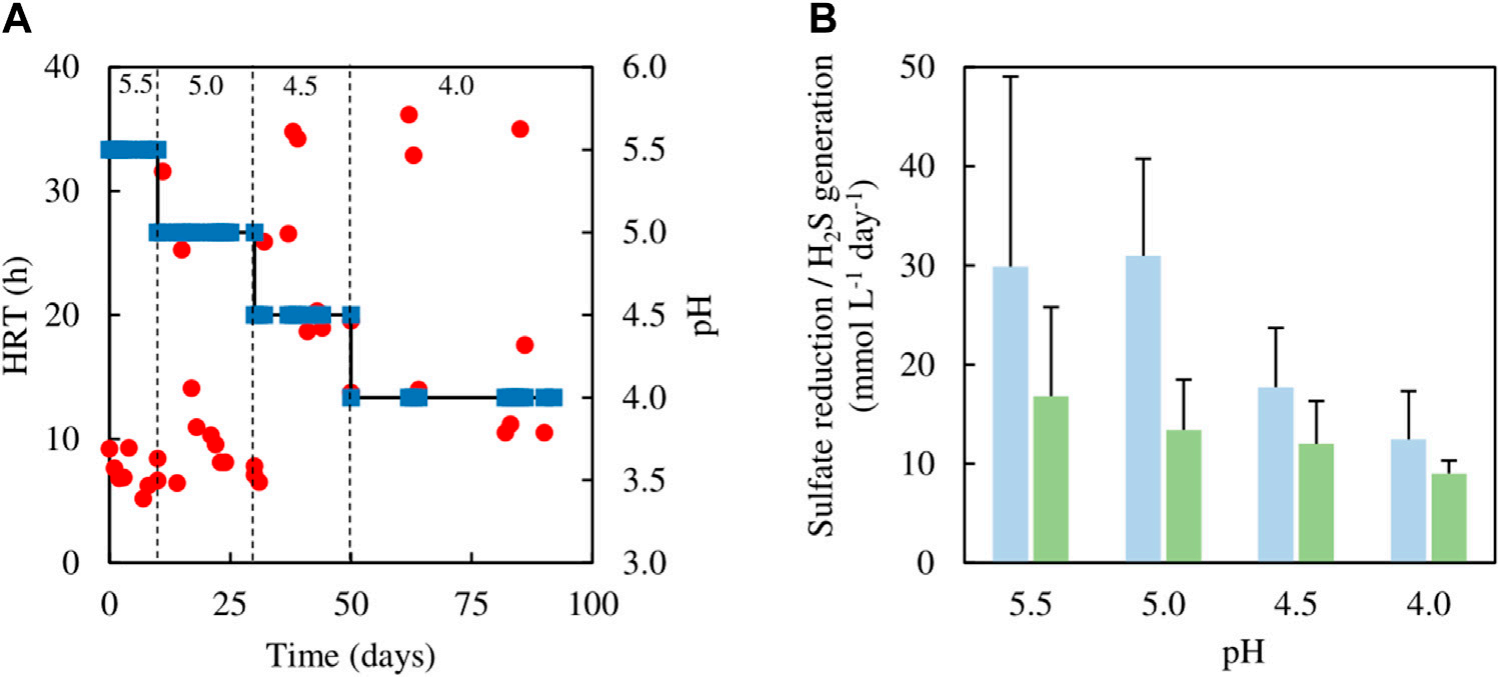
**(A)** Changes in HRTs (●) at different operational pH values (■) of the sulfidogenic bioreactor fed with ethanol alone (equivalent to 8 mM), and **(B)** variations in rates of sulfate reduction (dark green bars) and hydrogen sulfide generation (light green bars) in each bioreactor pH tested (*n* = 12). Samples were not analysed in the intervals between days 50–62 and days 64–82.

**FIGURE 9:**
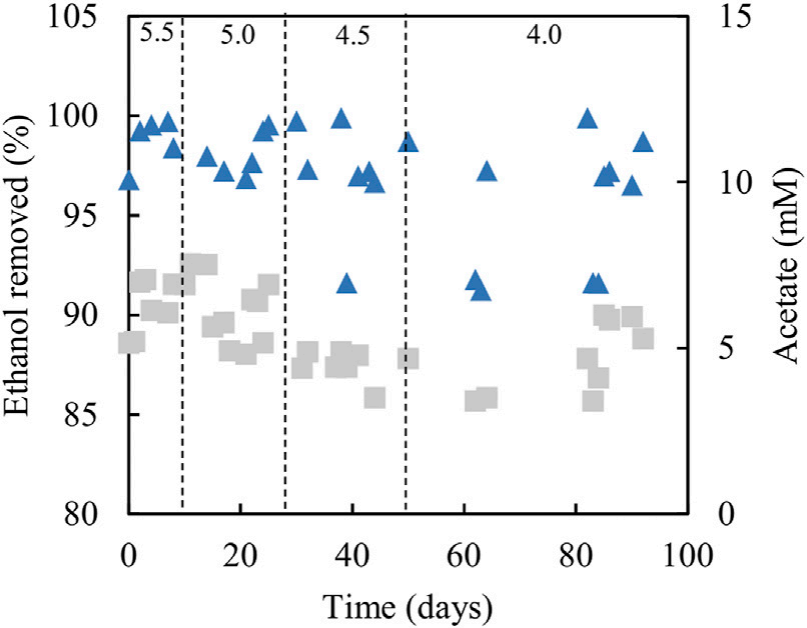
Differences in ethanol concentrations in influent and effluent liquors (▲), and concentrations of acetate/acetic acid in effluent liquors (■), during tests carried out with the sulfidogenic bioreactor using ethanol as the main electron donor. Samples were not analysed in the intervals between days 50–62 and days 64–82.

Analytical data from the sulfidogenic bioreactor with ethanol as the sole small molecular weight alcohol as electron donor at increasingly low pH values, are shown in Figures 8, 9. Decreasing the pH of the bioreactor incrementally by 0.5 of a pH unit on each occasion resulted in HRTs to increase but subsequently decrease (Figure 8A). Changing pH from 5.5 to 5.0 had no significant impact on rates of sulfidogenesis, but at lower pH values less hydrogen sulfide was generated (Figure 8B). Ethanol appeared to be used effectively (>95% removal) at all pH values tested (Figure 9). Acetate was generated throughout this experiment, and the general trend observed was that concentrations in the effluent liquors decreased in line with decreasing bioreactor pH, though there was considerable variation at pH 4.0 (Figure 9).

**FIGURE 10:**
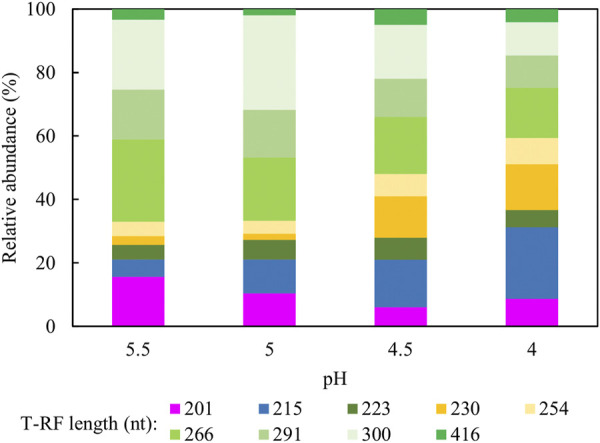
Terminal restriction enzyme fragment length polymorphism (T-RFLP) analysis (HaeIII digests) of amplified 16S rRNA genes of planktonic bacterial communities in the sulfidogenic bioreactor fed with ethanol alone, at different pH values. Shades of blue represent known sulfate-reducing *Firmicute* (215 nt, *D. acididurans*), shades of yellow represent non-sulfidogens (230 nt, *Actinobacterium* AR3; 253 nt, *At. ferrooxidans*), shades of green represent bacterial clones identified as probable non-sulfidogens and the magenta bars represent clone SRB7 (201 nt; the sulfidogenic Gram-negative bacterium *Dv. desulfuricans*).

Figure 10 shows changes in relative abundance of the planktonic bacterial community of the sulfidogenic bioreactor when its pH was lowered gradually from pH 5.5 to pH 4.0. In phase E100_5.5, the bacterium represented by clone SRB8 (266 nt; 89% related to *Bacteroides caecigallinarum*) accounted for ∼25% of the relative abundance of bacterial population in the bioreactor. *Desulfovibrio desulfuricans* (201 nt; clone SRB7) showed a higher relative abundance at pH 5.5 (∼16%) which declined when the pH was lowered (∼9% at pH 4.0). The opposite trend was observed for *D. acididurans* ^T^ (215 nt). An increase in the relative abundances of both *At. ferrooxidans* (254 nt) and the *Actinobacterium* AR3 (230 nt) occurred when pH was incrementally lowered. Other T-RFs did not show major differences in response to lowering the pH of the bioreactor.

## Discussion

The application of technologies that use SRB to remediate metal-contaminated wastewaters has been the focus of a large amount of research and development during recent times. Experiments described in this work examined the effects of combining three small molecular weight alcohols on the performance of a sulfidogenic bioreactor operated mostly at moderately acidic (pH 5.5) conditions.

The addition of methanol to the feed liquor had an initial negative impact on the performance of the sulfidogenic bioreactor which was greater than could be accounted for by the fact that the molar ratio of glycerol was concurrently decreased. The implication therefore was that methanol was toxic to at least some of the sulfidogens that were using glycerol as electron donor. Methanol addition also resulted in the detection of bacteria that had previously not been found in the bioreactor, most notably the sulfate-reducer *Dv. desulfuricans*. This genus has previously been reported to be able to couple the oxidation of methanol to the reduction of sulfate (Hard and Babel, 1997). The original material used to inoculate the low pH sulfidogenic bioreactor contained an undefined microbial mat sampled at a mine drainage stream in Spain (Rowe et al., 2007) and the implication is that *Dv. desulfuricans* had survived within the bioreactor community since that time, but in numbers that were below the limits of the method used to detect bacteria. *Dv. desulfuricans* was found in relatively large relative abundance throughout the entire time that methanol was included in the feed liquor, and its appearance seemingly helped to overcome the apparent toxicity of methanol to other sulfidogens in the microbial community. The net bioreactor performance, resulting in rates of sulfate reduction/sulfide production, increased when the carbon equivalent ratio of methanol to glycerol was increased from 10% to 50%. However, beyond this (phase M75) rates of sulfidogenesis decreased once again. This could have again been due to methanol toxicity, though the concentration used in phase M75 (12 mM) was only slightly greater than that used elsewhere (10 mM) to cultivate two other species of *Desulfovibrio* (*Dv. salixigens* and *Dv. carbinolicus*; Hard and Babel, 1997).

Several studies have reported ethanol utilization by SRB at circum-neutral pH (e.g., Bryant et al., 1977; Suzuki et al., 2007) and the use of this alcohol to fuel biosulfidogenesis (at pH 6–9) to remediate acid mine drainage (Barnes et al., 1991; Zagury et al., 2006). However, relatively little is known about sulfidogenesis at low pH with ethanol as an electron donor. The initial tests (all conducted at pH 5.5) confirmed that ethanol could be readily accessed by the microbial consortium used in the present study. Indeed, increasing the relative proportions of ethanol in the feed increased rates of sulfidogenesis (Figure 4B). However, one of the consequences of using ethanol was that concentrations of acetate in the effluents increased, to the point at which (phase E100) all of the ethanol removed appeared to be due to incomplete oxidation to acetate and CO_2_. The indigenous microbial community was also modified by increasing the relative amounts of ethanol in the feed, with the non-sulfidogens *Actinobacterium* AR3 and *At. ferrooxidans* becoming far less abundant in phases E50 - E100 (corresponding to 5–8 mM ethanol in the feed liquors). *Dv. desulfuricans* was detected throughout the time that ethanol was used, and this sulfidogen is known to metabolise ethanol as well as glycerol (Alico and Liegey, 1966; Nagpal et al., 2000). Ethanol continued to be used as electron donor for sulfidogenesis when the bioreactor pH was decreased incrementally. Data in Figure 8B suggest that rates of sulfate reduction decreased as the pH was lowered, but the bar graphs shown include all of the data from each of the operational phases. As Figure 8A illustrates, HRTs tended to decrease with time within each phase, reflecting the adaptation of the microbial community to increased acidity. When data from phase E100_4.0 are separated into those from between days 50–62 and days 82–92 of the 42 days of phase E100_4.0, they show HRTs of ∼30 and 16 h and SRRs of 9 and 14 mmol L^−1^ day^−1^, respectively. It is interesting to note, however, that a similar sulfidogenic bioreactor operated at pH 4.0 with 5 mM glycerol as the sole small molecular weight alcohol as electron donor was reported to have SRRs of ∼18 mmol L^−1^ day^−1^ (Santos and Johnson, 2017).

These experiments have shown that all three alcohols tested can be used to fuel microbial sulfidogenesis at moderately low pH (and at pH 4.0 in the case of ethanol). Each of them, however, has merits and detractions. A major advantage of methanol is that this C_1_ compound is oxidised completely to CO_2_ with no generation of acetate as a secondary waste material. This could eliminate the potential toxicity of the latter, which exists predominantly as acetic acid at pH < 4.75 (the *p*K_a_ of acetic acid/acetate). However, the apparent toxicity of methanol itself to at least some of the sulfate-reducing bacteria in the bioreactor means that its concentration would have to be maintained at a relatively low level (certainly in terms of carbon equivalents) for effective sulfate reduction. Ethanol, in contrast, did not appear to be toxic to the sulfidogens in the microbial community to a detectable extent (though some non-sulfidogens were impacted) but its main detraction was that it was mostly all oxidised incompletely, thereby greatly reducing its cost-effectiveness when being considered as the electron donor in a commercial operation. Comparing the reactions shown in Eqs 1–5, it is apparent that incomplete oxidation of ethanol coupled to sulfate reduction (2 ethanol:1 sulfate) is less effective than when methanol is used (2 methanol:1.5 sulfate) whereas corresponding figures for glycerol are 2:3.5 (complete oxidation) and 2:1.5 (incomplete oxidation). Other factors that need to be considered are: 1) the different volatilities of the three alcohols, as indexed by their different vapour pressures at 20°C (methanol 11.9 kPa, ethanol 5.95 kPa and glycerol 0.00001 kPa) which could lead to greater loss of the smaller molecular weight alcohols at higher temperatures where (as in the current case) there is continuous throughput of gas, and 2) global prices. In May 2022 these were (USD per MT): $598 for methanol (Methanex Corporation, 2022), $877 for ethanol (Trading Economics, 2022) and $473 for glycerol (Oleoline Ltd, 2020).

The bioreactor community contained bacteria that are not known sulfidogens. Their presence in low pH sulfidogenic bioreactors and explanations of how they were able to persist in these continuous flow systems has been reported and discussed elsewhere (e.g., Ñancucheo and Johnson, 2012; Santos and Johnson, 2017). *Actinobacterium* AR3 is able to ferment glycerol to acetate (Ñancucheo and Johnson, 2012) as are two of the bacteria detected in the clone bank analysis: *Clostridium beijerinckii* (Sanguanchaipaiwong and Leksawasdi, 2017) and *Propionicimonas paludicola* (Akasaka et al., 2003). Their impact on glycerol consumption appeared, however, to be minor, as stoichiometric analysis suggested that most if not all of the electrons obtained from the oxidation of glycerol, ethanol and methanol were used to reduce sulfate. The relative abundance of the two main sulfidogens detected in T-RFLP analysis changed not only with the ratios of alcohols in the feed liquors but also with the pH of the bioreactor. For example, the relative abundance of *Dv. desulfuricans*, which has been reported to grow optimally at pH 7.8 (Alico and Liegey, 1966) declined as the bioreactor pH was lowered, while that of *D. acididurans* (a sulfidogen that has been reported to grow well at pH 4.0; Sánchez-Andrea et al., 2015) increased. Molecular analyses of the bacterial population immobilised on glass beads were not performed in this study to avoid any disturbance to the biofilm bed. Variations in composition of planktonic and attached bacterial populations in similar low pH sulfidogenic bioreactor have been described elsewhere (Santos and Johnson, 2018).

The work described herein has confirmed that, beside glycerol, at least two other small molecular weight alcohols can serve as electron donors to fuel sulfidogenesis in moderately low pH bioreactors. Further experimental work needs to be carried out, for example to determine the lowest pH for methanol utilisation, as the sole methanol-oxidising sulfidogen detected in the present study (*Dv. desulfuricans*) was only tested in a bioreactor maintained at pH 5.5, and far lower pH values have been tested successfully (pH 2.2) with sulfidogenic *Firmicutes* as the bacteria responsible for reducing sulfate to hydrogen sulfide (Ñancucheo and Johnson, 2012). More crude potential feed material, such as those from biodiesel production, might ultimately prove to be the most cost-effective, though other components (e.g., “soap”) would have to be checked for their possible toxicities to the sulfidogenic population.

## Data Availability

The original contributions presented in the study are included in the article/supplementary material, further inquiries can be directed to the corresponding author.
